# Fine Mapping of *Carbon Assimilation Rate 8*, a Quantitative Trait Locus for Flag Leaf Nitrogen Content, Stomatal Conductance and Photosynthesis in Rice

**DOI:** 10.3389/fpls.2017.00060

**Published:** 2017-01-31

**Authors:** Shunsuke Adachi, Kazuaki Yoshikawa, Utako Yamanouchi, Takanari Tanabata, Jian Sun, Taiichiro Ookawa, Toshio Yamamoto, Rowan F. Sage, Tadashi Hirasawa, Junichi Yonemaru

**Affiliations:** ^1^Department of Biological Production Science, Graduate School of Agriculture, Tokyo University of Agriculture and TechnologyFuchu, Japan; ^2^Institute of Global Innovation Research, Tokyo University of Agriculture and TechnologyFuchu, Japan; ^3^Precursory Research for Embryonic Science and Technology, Japan Science and Technology AgencyKawaguchi, Japan; ^4^Institute of Crop Science, National Agriculture and Food Research OrganizationTsukuba, Japan; ^5^Department of Frontier Research, Kazusa DNA Research InstituteKisarazu, Japan; ^6^Rice Research Institute, Shenyang Agricultural UniversityShenyang, China; ^7^Department of Ecology and Evolutionary Biology, University of TorontoToronto, ON, Canada

**Keywords:** leaf nitrogen content, *Oryza sativa*, photosynthesis, quantitative trait locus, RuBP regeneration, stomatal conductance

## Abstract

Increasing the rate of leaf photosynthesis is one important approach for increasing grain yield in rice (*Oryza sativa*). Exploiting the natural variation in CO_2_ assimilation rate (*A*) between rice cultivars using quantitative genetics is one promising means to identify genes contributing to higher photosynthesis. In this study, we determined precise location of *Carbon Assimilation Rate 8* (*CAR8*) by crossing a high-yielding *indica* cultivar with a Japanese commercial cultivar. Fine mapping suggested that *CAR8* encodes a putative Heme Activator Protein 3 (OsHAP3) subunit of a CCAAT-box-binding transcription factor called OsHAP3H. Sequencing analysis revealed that the *indica* allele of *CAR8* has a 1-bp deletion at 322 bp from the start codon, resulting in a truncated protein of 125 amino acids. In addition, *CAR8* is identical to *DTH8/Ghd8/LHD1*, which was reported to control rice flowering date. The increase of *A* is largely due to an increase of RuBP regeneration rate via increased leaf nitrogen content, and partially explained by reduced stomatal limitation via increased stomatal conductance relative to *A*. This allele also increases hydraulic conductivity, which would promote higher stomatal conductance. This indicates that *CAR8* affects multiple physiological aspects relating to photosynthesis. The detailed analysis of molecular functions of *CAR8* would help to understand the association between photosynthesis and flowering and demonstrate specific genetic mechanisms that can be exploited to improve photosynthesis in rice and potentially other crops.

## Introduction

Rice (*Oryza sativa*) is one of the most valuable crops in the world, both in terms dollar value and contribution to the human food supply (FAO, 2015). Increasing its yield is a major challenge for improving global food security (Khush, 2013) and could be achieved by increasing the rate of net CO_2_ assimilation rate in individual leaves (*A*) (Long et al., 2006; Murchie et al., 2009). While photosynthetic improvement often emphasizes improving specific known traits within the photosynthetic apparatus (Suzuki et al., 2007; Takahara et al., 2010; von Caemmerer and Evans, 2010), or through introducing novel photosynthetic pathways such as the C_4_ pathway (Kajala et al., 2011; http://C4rice.irri.org), analysis of quantitative trait locus (QTL) through crossing experiments provide the opportunity to identify novel genetic elements that control photosynthetic performance in existing rice cultivars (Flood et al., 2011).

Most agronomic traits including *A* are controlled by multiple genetic factors, such traits are known as quantitative traits. QTL analyses can provide associations between quantitative traits and molecular markers (Tanksley, 1993). To conduct a QTL analysis, phenotypic values of interest are quantified in a segregating population whose genotypes have been determined by DNA markers. In rice, the complete genome sequence is available and many DNA markers have been identified (International Rice Genome Sequencing Project, 2005). Several advanced populations, including backcrossed inbred lines and chromosome segment substitution lines, have been developed to facilitate the QTL investigations in rice (Yamamoto et al., 2009). As a result, many genes associating with important agronomic traits have been identified using QTL methods (Yamamoto et al., 2014).

Wide variations in *A* among rice cultivars have been described (Takano and Tsunoda, 1971; Cook and Evans, 1983; Yeo et al., 1994; Kanemura et al., 2007; Jahn et al., 2011), and several QTL underlying this variation have been identified in populations derived from crosses between *japonica* and *indica* cultivars (Teng et al., 2004; Hu et al., 2009; Takai et al., 2010) and between *japonica* and *indica*/*japonica* cultivars (Gu et al., 2012). However, there is only one report that identified a causal gene controlling photosynthetic variation among rice cultivars (Takai et al., 2013). To understand the whole picture of the genetic control of *A* and to apply it in breeding aimed at increasing rice grain yield, it is necessary to identify the causal genes and understand their physiological aspects.

The CO_2_ assimilation rate in C_3_ species is considered to be limited by ribulose 1,5-bisphosphate (RuBP) carboxylation capacity of Rubisco or the RuBP regeneration capacity (Farquhar et al., 1980). Under low CO_2_ concentration and light-saturated conditions, *A* is commonly limited by the RuBP carboxylation capacity, while it is limited by RuBP regeneration capacity under elevated CO_2_ concentration and light-saturated conditions. The RuBP regeneration capacity reflects the capacity of electron transport, the Calvin cycle, and under high CO_2_ concentration, the ability of starch and sucrose synthesis to release inorganic phosphate (Sharkey, 1985). The CO_2_ diffusion from air into leaves is also important determinant of *A* (Farquhar and Sharkey, 1982). In healthy leaves, stomatal conductance (*g*_s_) is regulated to track the value of *A* such that the intercellular CO_2_ concentration (*C*_i_) and the ratio of intercellular to ambient CO_2_ (*C*_i_*/C*_a_) vary little as *A* increases (Farquhar and Sharkey, 1982). In contrast, Kusumi et al. (2012) shows that the increase in *g*_s_ relative to *A* can enhance *A* and *C*_i_*/C*_a_ in a rice mutant with a defective anion channel in the guard cells. This suggested that we should consider both the stomatal control and the enzymatic control of the photosynthetic apparatus to know the physiological reasons relating to the difference in *A*.

During grain filling, the flag leaf is the most important leaf in the rice canopy because its position at the top of the canopy ensures maximum light availability and it has greater photosynthetic capacity than leaves lower in the canopy. In our previous research, we used chromosome segment substitution lines derived from “Habataki,” a high-yielding *indica* cultivar with high *A*, and the *japonica* variety “Koshihikari,” the most popular cultivar in Japan with lower *A*, to identify four QTLs affecting *A* in flag leaves (Adachi et al., 2011, 2014). One of the four QTLs was identified at ~1.2 Mb region on the short arm of chromosome 8 (Adachi et al., 2011). According to the rice annotation database, 124 genes are predicted in this region (Sakai et al., 2013, http://rapdb.dna.affrc.go.jp). To determine gene responsible for the increase in *A*, fine-scale mapping is required. In this study, we examined a region in the QTL that correlates with the increase in *A*, which we term *Carbon Assimilation Rate 8 (CAR8)*. Our objective is to identify the gene underlying *CAR8* via fine mapping and to evaluate the physiological mechanism by which it increases *A*.

## Materials and methods

### Growth conditions

We grew rice plants in three conditions—paddy fields, outdoors in pots, and in a controlled-environment cabinet in pots. We used the plants grown in paddy fields for QTL mapping, the plants grown outdoors in pots for evaluating the physiological effect of *CAR8* on *A*, and the plants grown in a controlled environment cabinet in pots for evaluating the hydraulic conductance and root surface area. Plants in a paddy field were grown at the National Institute of Agrobiological Sciences in Tsukuba, Japan (36°03′N, 140°11′E). Seedlings at the fifth-leaf stage were transplanted (one plant per hill) into the field (alluvial clay loam). Each line was planted in a single row of 12 hills (18 cm between hills and 30 cm between rows) and fertilized with 56kg N, 176kg P_2_O_5_, and 56 kg K_2_O ha^−1^ with no top dressing was applied. Plants in pots were grown outdoors in 12-L pots filled with a 1:1 (v/v) mixture of paddy soil (alluvial clay loam) and upland soil (diluvial volcanic ash) at a density of three hills per pot (three plants per hill). Fertilizer (1.0 g each N, P_2_O_5_, and K_2_O per pot) was applied at planting, and additional fertilizer (0.3 g N per pot) was applied at 69 and 85 days after sowing (DAS). Plants grown in a controlled-environment cabinet (14.5 h light/9.5 h dark; 28°C for 12 h and 24°C for 12 h) were in 3-L pots filled with a flooded, granular culture soil. The relative humidity was 60%; the photosynthetic photon flux density (PPFD) at the top of the canopy was 500 μmol photons m^−2^ s^−1^. The soil contained 1.2 g N, 3.2 g P_2_O_5_, and 1.8 g K_2_O per pot.

### Plant materials for QTL mapping

**Figure 1:**
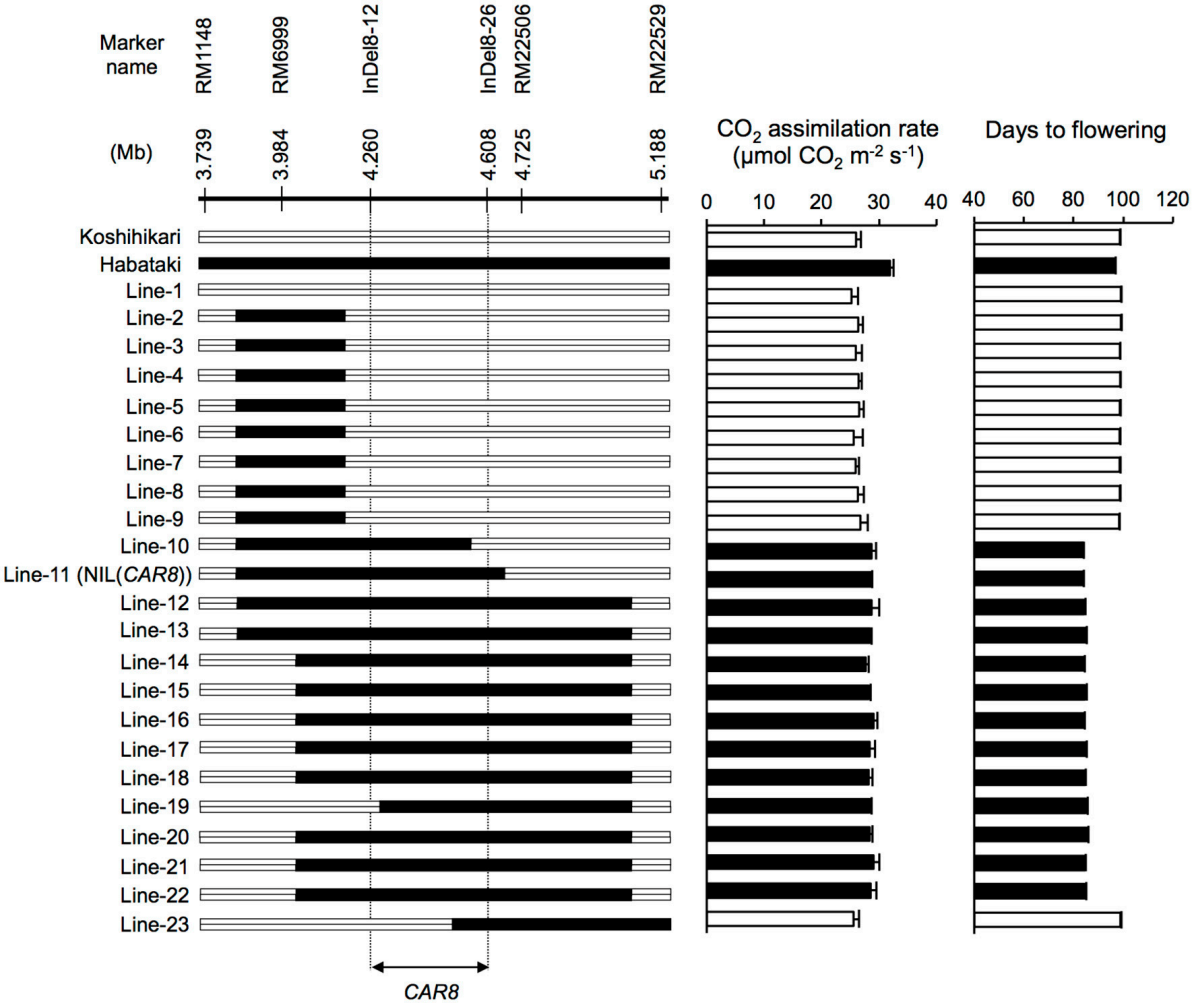
**Substitution mapping of ***CAR8*** using homozygous recombinant lines (BC_**5**_F_**6**_)**. Molecular markers are shown from the short arm **(left)** to the long arm **(right)** of chromosome 8. White segments, homozygous for “Koshihikari” alleles; black segments, homozygous for “Habataki” alleles. Field-grown plants were used. CO_2_ assimilation rate of flag leaves was measured at an ambient CO_2_ concentration of 370 μmol mol^−1^, a PPFD of 2000 μmol photons m^−2^ s^−1^, a leaf-to-air vapor pressure difference of 1.3–1.6 kPa, and an air temperature of 30°C. Black bars in graphs indicate significant difference from “Koshihikari” at the 5% level by Dunnett's test. Error bars indicate SD (*n* = 3).

**Figure 2:**
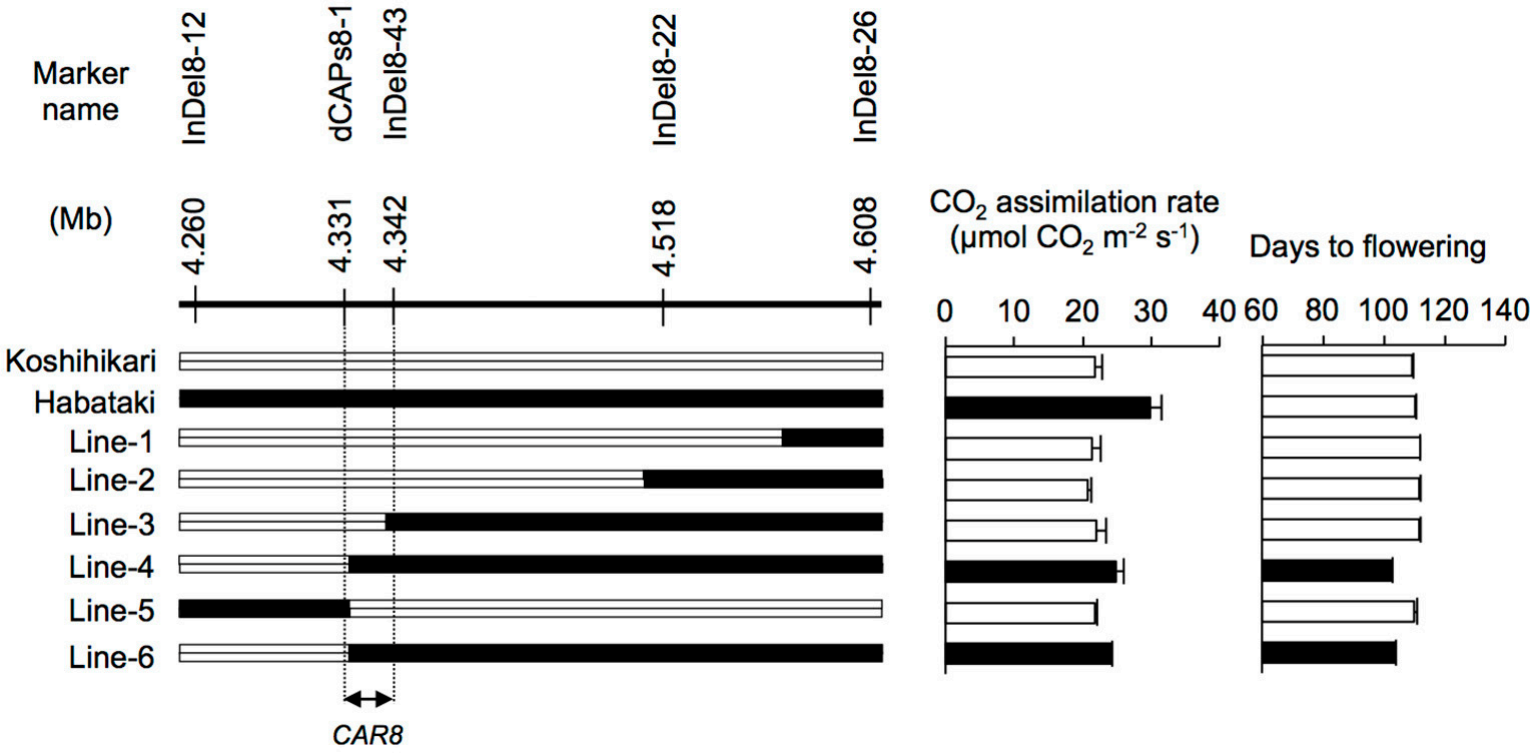
**Fine mapping of ***CAR8*** using homozygous recombinant lines (BC_**5**_F_**7**_)**. Molecular markers are shown from the short arm **(left)** to the long arm **(right)** of chromosome 8. White segments, homozygous for “Koshihikari” alleles; black segments, homozygous for “Habataki” alleles. Field-grown plants were used. CO_2_ assimilation rate of flag leaves was measured at an ambient CO_2_ concentration of 370 μmol mol^−1^, a PPFD of 2000 μmol photons m^−2^ s^−1^, a leaf-to-air vapor pressure difference of 1.3–1.6 kPa, and an air temperature of 30°C. Black bars in graphs indicate significant difference from “Koshihikari” at the 5% level by Dunnett's test. Error bars indicate SD (*n* = 3).

*CAR8* mapping was carried out using self-pollinated progenies derived from a BC_5_F_4_ population (912 plants) of a “Koshihikari” × “Habataki” cross with “Koshihikari” as the recurrent parent. They have a single heterozygous region in chromosome 8 and most other regions were homozygous for “Koshihikari” alleles. We selected 23 plants from the BC_5_F_4_ population and used homozygous BC_5_F_6_ generation for phenotyping (Figure 1). The near isogenic line NIL(*CAR8*) was also selected from the BC_5_F_6_ generation. Subsequently, fine mapping was carried out using self-pollinated progenies derived from a BC_5_F_5_ population (144 plants) of the same “Koshihikari” × “Habataki” cross. We selected 6 plants from the BC_5_F_5_ generation and used homozygous BC_5_F_7_ generation for the phenotyping (Figure 2). Molecular markers used for mapping are listed in Table S1. These plants were grown in the paddy field. For both experiments, a randomized block design (three replicates) was used and 4~6 plants were evaluated in each replicate.

### Gas exchange and nitrogen measurements

Leaf gas exchange was measured with a portable gas-exchange system (LI-6400; LI-COR, Lincoln, NE, USA) and 2 × 3 cm cuvette with an LED irradiation source (LI-6400- 02B; LI-COR). The uppermost fully expanded leaves were used for the measurements before heading, and flag leaves after heading. *A* and *g*_s_ were measured at an ambient CO_2_ concentration of 370 μmol mol^−1^, PPFD of 2000 μmol photons m^−2^ s^−1^, a leaf-to-air vapor pressure difference of 1.3–1.6 kPa, and a leaf temperature of 30°C. Plants were examined from 08:30 to 11:30, when the photosynthetic rate was close to the daily maximum (Hirasawa and Ishihara, 1992). The CO_2_ assimilation rate vs. *C*_i_ was examined at a light intensity of 2000 μmol photons m^−2^ s^−1^ and a leaf temperature of 30°C at full heading stage by changing the ambient CO_2_ concentration. To prevent potential leaks, we sealed the gaskets with vacuum grease. Rubisco-limited photosynthesis (*A*_c_) was calculated from Farquhar et al. (1980) as:

$$\begin{array}{l} A_{\text{c}}=\left[V_{\text{cmax}}\left({\text{C}}_{\text{i}}-\Gamma^{*}\right)\right]/\left[{\text{C}}_{\text{i}}+{\text{K}}_{\text{c}}\left(1+{\text{O/K}}_{o}\right)\right]-{\text{R}}_{\text{d}}, \end{array}$$

where Γ^*^ (μmol mol^−1^) is the CO_2_ compensation point in the absence of day respiration, *K*_c_ (μmol mol^−1^) and *K*_o_ (mmol mol^−1^) are the Michaelis constants for CO_2_ and O_2_ respectively, and *R*_d_ (μmol mol^−1^) is the day respiration rate. Photosynthetic rate limited by RuBP regeneration capacity (*A*_r_) is calculated as;

$$\begin{array}{l} {\text{A}}_{\text{r}}=\left[{\text{J}}_{\text{cmax}}\left({\text{C}}_{\text{i}}-\Gamma^{*}\right)\right]/\left(4{\text{C}}_{\text{i}}+8\Gamma^{*}\right)-{\text{R}}_{\text{d}}, \end{array}$$

The *K*_c_, *K*_o_, and Γ^*^ at 30°C were calculated from the data of Makino et al. (1988) using the Arrhenius function described by von Caemmerer (2000). To convert the *K*_c_ and *K*_o_ from concentrations to partial pressures, solubilities of 0.0334 mol L^−1^ bar^−1^ for CO_2_ and 0.00126 mol L^−1^ bar^−1^ for O_2_ were used (von Caemmerer, 2000). *A*/*C*_i_ response curves were analyzed using the mathematical model developed by Sharkey et al. (2007) and the data were automatically fitted with the model fitting utility based on a Microsoft Excel program (http://www.blackwellpublishing.com/plantsci/pcecalculation/).

Immediately after the measurements of photosynthesis, 30-mm-long segment was cut from the center of the leaf of measured plants and stored at −80°C. The leaves were then dried at 80°C for 24 h and the nitrogen content was assayed using with a CN analyzer (MT700 Mark II, Yanako, Kyoto, Japan).

### Determination of stomatal density and pore length

The middle part of flag leaves was fixed in solution containing (v/v) 5% formalin, 5% acetic acid, and 45% ethyl alcohol in distilled water. Abaxial and adaxial surfaces of the fixed leaves were photographed under a scanning electron microscope (TM3030; Hitachi, Tokyo, Japan). Stomatal number was counted using a touch screen (Flexscan T2351W; Eizo, Ishikawa, Japan) connected to a computer that was installed with original computer software that senses the number of contacts. Length of stomatal pores was analyzed with ImageJ software (National Institutes of Health, Bethesda, MD, USA).

### Determination of hydraulic conductance and hydraulic conductivity of plants

The hydraulic conductance from roots to leaves (*C*_p_, 10^−8^ m^3^ s^−1^ MPa^−1^) was calculated as *U*_w_/(Ψ_s_ − Ψ_l_; Hirasawa and Ishihara, 1991), where *U*_w_ (10^−8^ m^3^ s^−1^) is the water uptake rate of the whole plant, Ψ_s_ (MPa) is the water potential of the soil immediately outside the root, and Ψ_l_ (MPa) is the average water potential of the uppermost three leaves. Since plants were submerged the water potential of the soil solution, Ψ_s_ was regarded as 0. Plants grown in 3-L pots were used. Measurements were made in a controlled-environment cabinet [air temperature, 28°C; air vapor pressure deficit (VPD), 1.5 kPa; PPFD at the top leaves, 900 μmol m^−2^ s^−1^]. *U*_w_ was determined from the rate of weight loss of the pot over 20 min after a steady state had been reached. To prevent evaporation from the surface of the pot, the top was covered with polystyrene foam and the gap between the foam and the stem was sealed with oil clay. After measurement of *U*_w_, Ψ_l_ was measured in a pressure chamber (model 3005; Soil Moisture Equipment, Santa Barbara, CA, USA). The transpiration rate and *g*_s_ do not influence *C*_p_ when the transpiration rate is high (Fiscus, 1975; Hirasawa and Ishihara, 1991; Stiller et al., 2003). The *U*_w_ per leaf area was sufficiently high (>2.0 mmol m^−2^ s^−1^) to eliminate the effect of the difference in water uptake rate on *C*_p_. After roots had been washed gently in water, root surface area (*S*_r_) was measured with an image analyzer (WinRHIZO REG V 2004b; Regent Instruments, Quebec, Canada). The hydraulic conductivity (*L*_p_,10^−8^ m s^−1^ MPa^−1^) was expressed as *C*_p_ per *S*_r_ (Steudle and Peterson, 1998).

### Response to the change of vapor pressure deficit

Plants grown outdoors in 12-L pots until full heading stage were moved to a controlled-environment cabinet (KG-50HLA; Koito Manufacturing Co. Ltd, Tokyo, Japan) at a PPFD and temperature at the flag leaf surface of 900 μmol photons m^−2^ s^−1^ and 30°C. Air humidity was modified in steps to generate a range of VPD values inside the cabinet. The temperature and humidity near the flag leaf were monitored with a thermo-hygro sensor (Climomaster model 6531; Kanomax, Osaka, Japan). *g*_s_ of the flag leaf was measured with the LI-6400 portable gas-exchange system after a steady state had been reached; the leaf chamber conditions were similar to those in the cabinet. After gas exchange measurements, water potential of each leaf was determined with the pressure chamber.

### Statistical analysis

For the fine mapping, Dunnett's test was applied in the mapping population. For comparisons of physiological traits, we analyzed ANOVA and least significant difference (LSD) test. All analyses were tested with JMP v.12 software (SAS Institute, Cary, NC, USA).

## Results

### Fine mapping of *CAR8*

**Figure 3:**
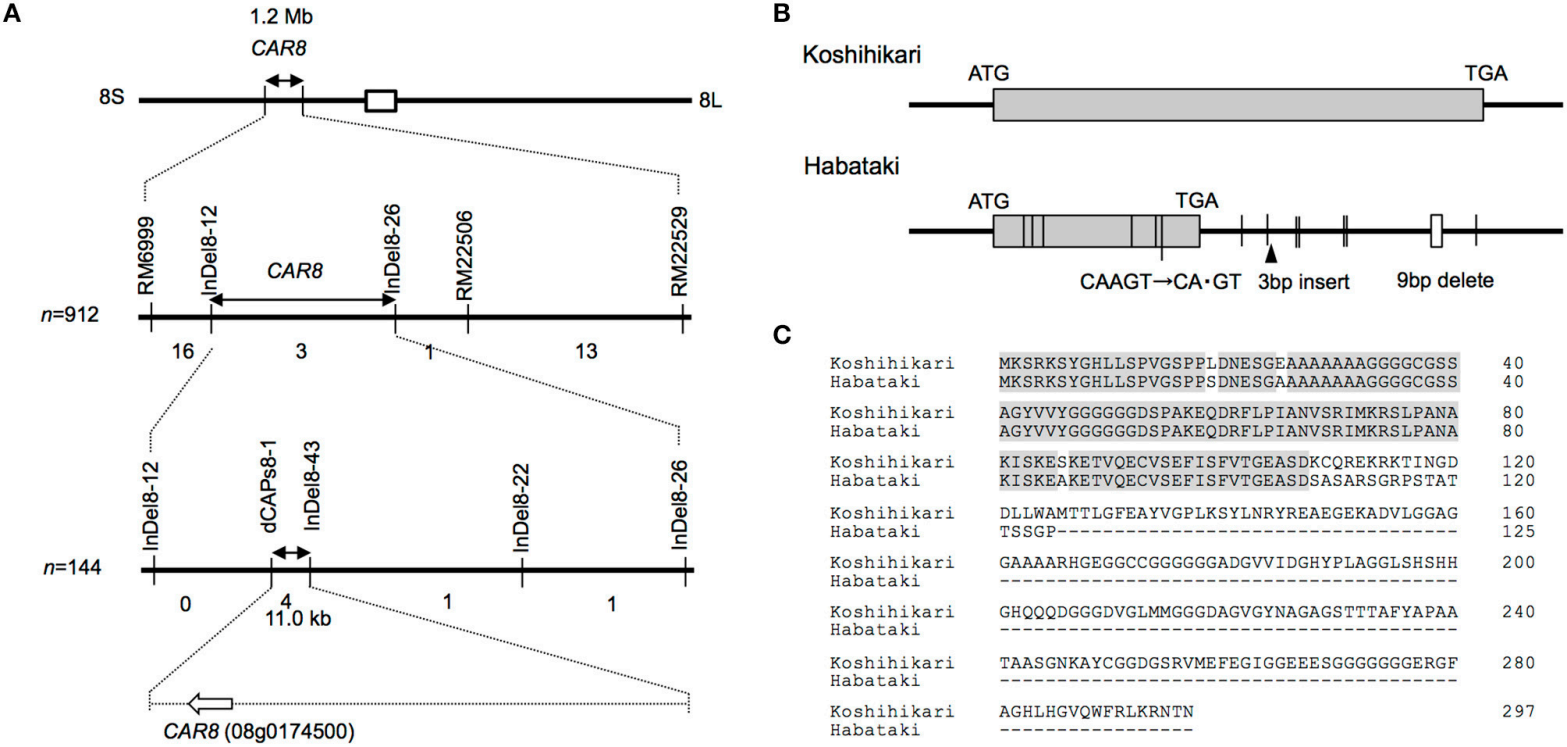
**Map-based cloning of ***CAR8***. (A)** Fine mapping of *CAR8*. The number of recombinants between molecular markers is indicated below the each line. **(B)** Structure of *CAR8*. The exon is shown as a gray box. Vertical lines without labels represent single-base substitutions between “Koshihikari” and “Habataki.” Small open boxes represent deletions. **(C)** Alignment of *CAR8* amino acid sequences.

Using homozygous recombinant lines derived from a cross between “Koshihikari” and “Habataki,” we conducted fine mapping of *CAR8* (Figures 1–3). These plants were grown in the paddy field. The *A* of the flag leaves was measured at full heading stage, which was 3–7 days after flowering, under light-saturated conditions and ambient CO_2_ concentration. Using lines of BC_5_F_6_ generation, we narrowed down the *CAR8* region to 348.3 kb between insertion-deletion (InDel) marker InDel8-12 and InDel8-26 on the short arm of chromosome 8 (Figure 1). Among lines of BC_5_F_7_ generation, two of the six lines showed higher *A*-values than “Koshihikari” (Figure 2). This enabled us to delimit the *CAR8* region to 11.0 kb between the derived cleaved amplified polymorphic sequence (dCAPS) marker dCAPS8-1 and InDel8-43 (Figure 3A). A single gene, Os08g0174500, was predicted in this region using the RAP-DB. Os08g0174500 encodes a Heme Activator Protein 3 (OsHAP3) subunit of CCAAT-box-binding transcription factor called OsHAP3H. This gene was same gene to *DTH8, Ghd8*, and *LHD1* (*days to heading 8, grain number, plant height and heading date 8*, and *Late Heading Date 1*), which have been reported to regulate heading date (Wei et al., 2010; Yan et al., 2011; Dai et al., 2012). The time to heading was 7–10 days shorter in two homozygous recombinant lines with higher *A* than in “Koshihikari” (Figures 1, 2). Sequence analysis of Os08g0174500 revealed that “Koshihikari” had a reading frame totaling 894 bp that encodes a protein of 297 amino acids. In “Habataki,” a 1-bp deletion at 322 bp from the initiation codon caused a frameshift and premature termination of translation, resulting in a truncated protein of 125 amino acids (Figures 3B,C).

### Photosynthesis response

The near isogenic line NIL(*CAR8*) was selected from the BC_5_F_6_ generation derived from a cross between “Koshihikari” and “Habataki” with DNA marker assisted selection (Figure 1). NIL(*CAR8*) carries a single chromosome segment of “Habataki,” which includes the *CAR8* region, in the genetic background of “Koshihikari” (Figure S1). The length of the substituted region in NIL(*CAR8*) was approximately 1.0 Mb.

**Figure 4:**
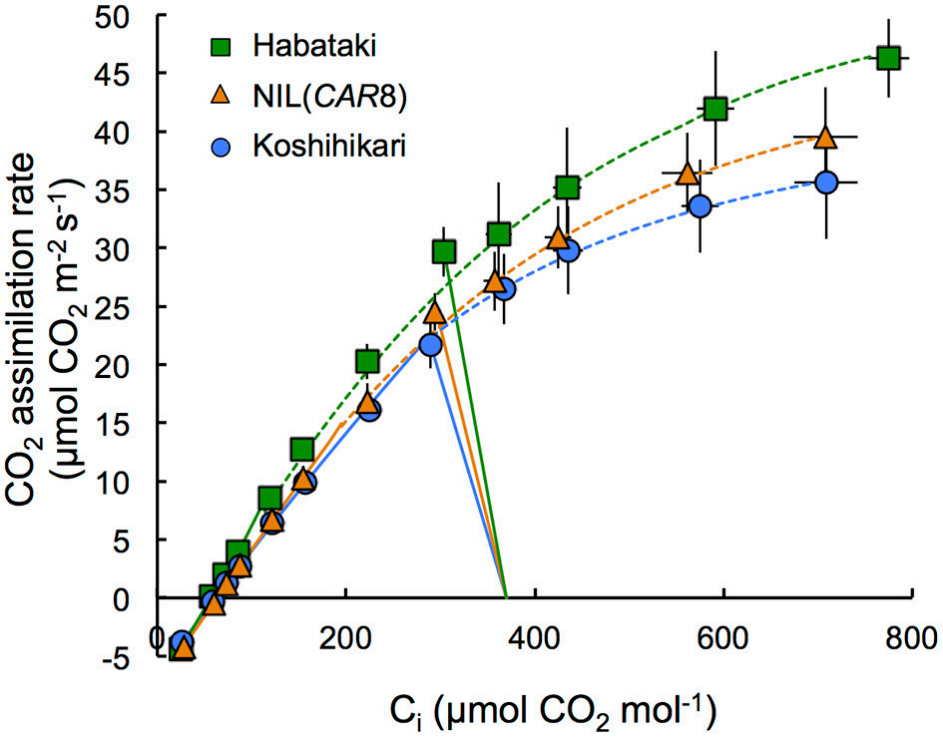
**Response of CO_**2**_ assimilation rate of flag leaves at full heading to intercellular CO_**2**_ concentration**. Plants of “Koshihikari” (circles), NIL(*CAR8*) (triangles), and “Habataki” (squares) were grown outdoors in 12-L pots. Leaf gas exchange was measured at a PPFD of 2000 μmol photons m^−2^ s^−1^ and an air temperature of 30°C. CO_2_ assimilation rate limited by RuBP carboxylation (solid line) and CO_2_ assimilation rate limited by RuBP regeneration (dotted line) were shown. Curve fitting was described in the Materials and Methods Section. The straight lines represent the measurement at ambient CO_2_ concentration of 370 μmol mol^−1^. Error bars indicate SD (*n* = 6).

Using plants grown outdoors in 12-L pots, we evaluated several traits that affect *A* (Table 1). At full heading stage, which was 2–4 days after flowering, *A* of the flag leaves in NIL(*CAR8*) at CO_2_ concentration of 370 μmol mol^−1^ was 16% higher than that of the flag leaves in “Koshihikari.” Leaf nitrogen content per leaf area (LNC_a_) and leaf nitrogen content per leaf dry weight (LNC_w_) in NIL(*CAR8*) were also higher than in “Koshihikari.” *V*_cmax_ and *J*_max_ estimated from *A*–*C*_i_ responses (Sharkey et al., 2007) were higher in NIL(*CAR8*) than in “Koshihikari.” *g*_s_ was higher in NIL(*CAR8*) than in “Koshihikari,” such that *C*_i_ and *C*_i_/*C*_a_ in NIL(*CAR8*) were also higher than those of “Koshihikari.” These values in “Habataki” were generally higher than those in NIL(*CAR8*), although the statistically significant differences were found in only *A*, LNC_a_ and *g*_s_. We also found that NIL(*CAR8*) had slightly higher *A* regardless of *C*_i_ values than “Koshihikari” in the *A*-*C*_i_ curve (Figure 4).

**Table 1:** **Photosynthetic parameters of flag leaves at the full heading stage**.

		**Koshihikari**	**NIL(*CAR8*)**	**Habataki**	**ANOVA**
*A*	μmol CO_2_ m^−2^ s^−1^	21.7 ± 0.73c	25.2 ± 1.5b	30.2 ± 1.7a	^***^
LNC_a_	g m^−2^	1.54 ± 0.09c	1.66 ± 0.10b	1.88 ± 0.12a	^**^
LNC_w_	mg g^−1^ DW	24.8 ± 1.3b	29.2 ± 3.4a	28.0 ± 0.6a	^**^
*V*_cmax_	μmol CO_2_ m^−2^ s^−1^	176.2 ± 23.2b	222.4 ± 39.9a	266.5 ± 23.7a	^**^
*J*_max_	μmol CO_2_ m^−2^ s^−1^	215.5 ± 32.2b	251.0 ± 23.3a	273.0 ± 24.0a	^**^
*g*_s_	mol H_2_O m^−2^ s^−1^	0.55 ± 0.10c	0.74 ± 0.06b	1.06 ± 0.10a	^***^
*C*_i_	μmol CO_2_ mol^−1^	289.4 ± 7.4b	297.4 ± 3.1a	303.3 ± 4.4a	^**^
*C*_i_/*C*_a_		0.78 ± 0.02b	0.80 ± 0.01a	0.82 ± 0.01a	^**^

*Plants were grown outdoors in 12-L pots. Leaf gas exchange was measured at an ambient CO_2_ concentration of 370 μmol mol^−1^, a PPFD of 2000 μmol photons m^−2^ s^−1^, a leaf-to-air vapor pressure difference of 1.3–1.6 kPa, and a leaf temperature of 30°C. Values are mean ± SD (n = 6)*.

** P < 0.01;

*** *P < 0.001. Values followed by the same letters indicate no significant difference among rice lines at P < 0.05 by LSD test. A, CO_2_ assimilation rate; LNC_a_, leaf nitrogen content per leaf area; LNC_w_, leaf nitrogen content per leaf dry weight; V_cmax_, maximum carboxylation rate; J_max_, maximum electron transport rate; g_s_, stomatal conductance; C_i_, intercellular CO_2_ concentration; C_i_/C_a_, ratio of intercellular to ambient CO_2_ concentration*.

**Figure 5:**
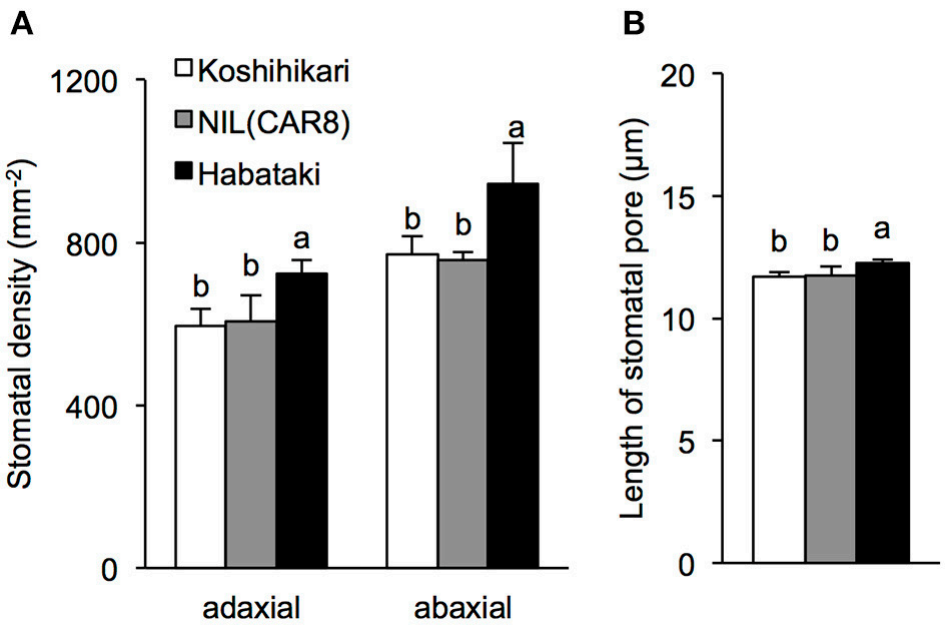
**Stomatal density (A) and stomatal pore length (B) in flag leaves of field-grown plants at full heading**. Error bars indicate SD (*n* = 3). Values followed by the same letters indicate no significant difference among rice lines at *P* < 0.05 by LSD test.

Values of *g*_s_ are affected by stomatal density, pore length, and aperture (Maruyama and Tajima, 1990; Ohsumi et al., 2007). Stomatal densities in the adaxial and abaxial epidermis were similar between NIL(*CAR8*) and “Koshihikari” (Figure 5A). There was no significant difference in the pore length between NIL(*CAR8*) and “Koshihikari” (Figure 5B). These values in “Habataki” were significantly higher than those in NIL(*CAR8*) and “Koshihikari.”

We monitored leaf gas exchange and LNC throughout the growth period (Table 2). The number of days from sowing to flowering was 100 in “Koshihikari,” 91 in NIL(*CAR8*), and 103 in “Habataki.” There was no difference in *A, g*_s_, LNC_a_, and LNC_w_ between “Koshihikari” and NIL(*CAR8*) at 47 and 67 days after sowing (DAS). At 95 DAS, *A, g*_s_, LNC_a_, and LNC_w_ in NIL(*CAR8*) were higher than those of “Koshihikari,” while only *g*_s_ was higher in NIL(*CAR8*) at 105 DAS. “Habataki” showed higher *A* than NIL(*CAR8*) from 67 to 105 DAS, which was accompanied by the higher *g*_s_ and in some cases higher LNC_a_ and LNC_w_.

**Table 2:** **CO_2_ assimilation rate (***A***), stomatal conductance (***g***_**s**_), and leaf nitrogen content (LNC) between 47 and 105 days after sowing (DAS)**.

	**Lines**	**DAS (Day)**				**ANOVA**		
		**47**	**67**	**95**	**105**	**Line**	**DAS**	**Line × DAS**
*A* μmol CO_2_ m^−2^ s^−1^	Koshihikari	33.5 ± 0.8a	18.4 ± 1.2b	21.7 ± 2.0c	21.7 ± 2.0b	^***^	^***^	^***^
	NIL(*CAR8*)	35.5 ± 1.6a	18.3 ± 1.6b	25.2 ± 1.5b	22.7 ± 1.5b			
	Habataki	33.6 ± 2.3a	22.2 ± 2.5a	29.8 ± 2.1a	29.5 ± 2.2a			
*g*_s_ mol H_2_O m^−2^ s^−1^	Koshihikari	0.67 ± 0.06b	0.42 ± 0.04b	0.55 ± 0.07c	0.55 ± 0.10c	^***^	^***^	^**^
	NIL(*CAR8*)	0.73 ± 0.07b	0.44 ± 0.08b	0.74 ± 0.06b	0.74 ± 0.03b			
	Habataki	0.88 ± 0.10a	0.67 ± 0.13a	1.02 ± 0.10a	0.98 ± 0.12a			
LNC_a_ g m^−2^	Koshihikari	2.33 ± 0.09a	1.13 ± 0.03b	1.53 ± 0.08b	1.54 ± 0.09b	^***^	^***^	^*^
	NIL(*CAR8*)	2.36 ± 0.12a	1.13 ± 0.03b	1.66 ± 0.10a	1.43 ± 0.10b			
	Habataki	2.42 ± 0.14a	1.22 ± 0.05a	1.84 ± 0.13a	1.88 ± 0.12a			
LNC_W_ mg g^−1^ DW	Koshihikari	45.6 ± 5.2a	22.7 ± 1.1a	24.5 ± 3.1b	24.8 ± 1.3b	^*^	^***^	NS
	NIL(*CAR8*)	44.9 ± 6.1a	21.8 ± 2.3a	29.2 ± 3.4a	24.7 ± 1.4b			
	Habataki	45.7 ± 1.8a	23.5 ± 1.6a	29.0 ± 1.8a	28.0 ± 0.6a			

*Plants were grown in 12-L pots. Gas exchange was measured on the uppermost fully expanded leaves at an ambient CO_2_ concentration of 370 μmol mol^−1^, a PPFD of 2000 μmol photons m^−2^ s^−1^, a leaf-to-air vapor pressure difference of 1.3–1.6 kPa, and a leaf temperature of 30°C between 08:30 and 11:30. LNC_a_, leaf nitrogen content per leaf area; LNC_w_, leaf nitrogen content per leaf dry weight. The number of days to heading was 100 for “Koshihikari,” 91 for NIL(CAR8), and 103 for “Habataki.” Nitrogen (0.3 g per pot) was applied at 69 and 85 DAS. Values are mean ± SD (n = 6)*.

* P < 0.05;

** P < 0.01;

*** *P < 0.001. NS, not significant at 0.05 probability level. Values followed by the same letters indicate no significant difference among rice lines at P < 0.05 by LSD test*.

### Hydraulic conductance

It is suggested that *g*_s_ is influenced by the hydraulic conductance of a plant (Brodribb and Holbrook, 2003). When we compared the plants grown in 3-L pots in a controlled-environment cabinet at the full heading stage, *C*_p_ in NIL(*CAR8*) was significantly higher than in “Koshihikari” (Table 3). The *C*_p_ in “Habataki” was much higher than that in NIL(*CAR8*). *C*_p_ can be divided in root surface area (*S*_r_) and hydraulic conductance per *S*_r_, i.e., hydraulic conductivity (*L*_p_) (Steudle and Peterson, 1998). NIL(*CAR8*) showed similar *S*_r_ but higher *L*_p_ in comparison with “Koshihikari,” while “Habataki” showed higher *S*_r_ but similar *L*_p_ in comparison with “Koshihikari.” We also determined that *A* and *g*_s_ in NIL(CAR8) were higher than those of “Koshihikari” (data not shown).

**Table 3:** **Hydraulic conductance from roots to leaves (***C***_**p**_), root surface area (***S***_**r**_), and hydraulic conductivity (***L***_**p**_) of plants grown in a controlled-environment cabinet**.

	***C*****_p_**	***S*****_r_**	***L*****_p_**
	**10^−8^ m^3^ s^−1^ MPa^−1^**	**m^2^**	**10^−8^ m s^−1^ MPa^−1^**
Koshihikari	0.128 ± 0.011c	0.079 ± 0.016b	1.66 ± 0.33b
NIL(*CAR8*)	0.191 ± 0.022b	0.081 ± 0.013b	2.39 ± 0.45a
Habataki	0.241 ± 0.006a	0.152 ± 0.024a	1.62 ± 0.24b
ANOVA	^***^	^**^	^*^

*The measurements were conducted at the vapor pressure deficit (VPD) of 1.5 kPa. C_p_ and S_r_ are expressed per stem. Values are mean ± SD (n = 4)*.

* P < 0.05;

** P < 0.01;

*** *P < 0.001. Values followed by the same letters indicate no significant difference among rice lines at P < 0.05 by LSD test*.

To assess relationship between leaf water status and *g*_s_, we compared the responses of transpiration rate (*T*), *g*_s_, and leaf water potential (Ψ_l_) to vapor pressure deficit (VPD) at the full heading stage with the plants grown in 12-L pots (Figure S2). In all genotypes, *T* increased and *g*_s_ and Ψ_l_ declined with increasing VPD. In NIL(*CAR8*) and “Habataki,” *T* and *g*_s_ were always higher than in “Koshihikari,” whereas Ψ_l_ was similar in all three genotypes irrespective to VPD conditions. These results indicate that NIL(*CAR8*) keeps Ψ_l_ at a certain level even though their *T* are significantly higher than “Koshihikari.”

### Grain yield

We examined the final grain yield of the plant grown in the paddy field (Figure S3). The brown rice yield in NIL(*CAR8*) was lower than that of “Koshihikari”, while the yield of “Habataki” was significantly higher than the others.

## Discussion

The understanding of genetic factors and their physiological aspects that control the natural variation of rice photosynthesis are important for future rice breeding aimed at increasing grain yield. In this study, we narrowed down the genetic region of *CAR8* located in the short arm of chromosome 8 and evaluated the physiological aspects of *CAR8*.

The result of the fine mapping suggests that the protein encoded by *CAR8* is a putative OsHAP3 subunit of the HAP complex, OsHAP3H. HAP complex binds to CCAAT box and act either as a transcription activator or as a repressor (Laloum et al., 2013). The HAP complex consists of three subunits: HAP2, HAP3, and HAP5 (Mantovani, 1999). Each of the HAP subunits is encoded by a single gene in yeast (*Saccharomyces cerevisiae*) and mammals (Mantovani, 1999), while in rice, the genome encodes 10 OsHAP2, 11 OsHAP3, and seven OsHAP5 subunits (Thirumurugan et al., 2008). *CAR8* might be identical to *DTH8/Ghd8/LHD1*, which was reported to control rice flowering date (Wei et al., 2010; Yan et al., 2011; Dai et al., 2012). According to the classification of Wei et al. (2010), the “Koshihikari” allele corresponds to type 1 and the “Habataki” allele to type 8. Under long-day conditions, the type 1 allele of *DTH8* negatively influenced the expression of *Early heading date 1* (*Ehd1*) and *Heading date 3a* (*Hd3a*), resulting in repression of flowering (Wei et al., 2010). Recently, it is revealed that *DTH8* binds to *Heading date 1* (*Hd1*), which represses the expression of *Ehd1* and control the heading date (Chen et al., 2014). The “Koshihikari” allele might suppress the expression of these genes and delay the heading date, while the allele of “Habataki” might not. Although it is well known that OsHAP3H regulates rice flowering, the association of this gene with photosynthesis has been little noticed. The detailed analysis of molecular mechanisms including complementation tests would contribute to understand how *CAR8* controls both photosynthesis and flowering.

Two hypotheses can generally explain an increase in *A* in C_3_ plants: (1) increase in the biochemical activity of the leaf photosynthetic machinery and (2) enhancement of CO_2_ diffusion from air into leaves (Farquhar and Sharkey, 1982). While we didn't find any difference in *A* during vegetative stage (i.e., at 47 and 67 DAS in Table 2), we found the higher *A* by 16% in NIL(*CAR8*) than that in “Koshihikari” at full heading stage. A higher abundance of photosynthetic proteins is indicated by a corresponding increase in LNC (Makino et al., 1984). We found that NIL(*CAR8*) had higher LNC_a_ and LNC_w_ than “Koshihikari” at the full heading stage (Table 1). Biochemically, the rate of photosynthesis is generally limited by either RuBP carboxylation capacity, or RuBP regeneration capacity in the broad sense (which would include Calvin cycle capacity and P_i_ regeneration in addition to electron transport rate; Farquhar et al., 1980; Sharkey, 1985). Yamori et al. (2011a) reported that the value of *A* in rice (cv. Notohikari) at an ambient CO_2_ concentration of 380 μmol mol^−1^ was limited by RuBP regeneration rate. We applied the theoretical analysis of our results using the Farquhar and von Caemmerer model (as modified by Sharkey et al., 2007) and found the *A* of NIL(*CAR8*) and “Kohishikari” at 370 μmol CO_2_ mol^−1^ tended to be limited by RuBP regeneration rate, although it is close to the limiting region of *V*_cmax_ (Figure 4). The increase of RuBP regeneration rate corresponds to increase of ~2.0 μmol m^−2^ s^−1^ of *A* at the ambient CO_2_ concentration when we calculated from the *A*-*C*_i_ curve. Hence, we conclude that the higher *A* in NIL(*CAR8*) than “Koshihikari” is mainly due to an enhanced *J*_max_ (Table 1). “Habataki” had even higher *J*_max_ and *A*, indicating “Habataki” includes additional QTL for enhanced *J*_max_.

A number of possibilities could explain how *J*_max_ is enhanced in NIL(*CAR8*) and “Habataki.” The simplest is that higher LNC_a_ and LNC_w_ in these lines provides more photosynthetic protein in both leaf area and leaf weight basis. This is probably the best explanation since both the *V*_cmax_ and *J*_max_ were increased, indicating an across the board enhancement of photosynthetic protein. With respect to *J*_max_, it has been suggested that the electron flow through the Cytochrome *b*_6_*/f* complex is a rate-limiting step for RuBP regeneration (Yamori et al., 2011b). Therefore, the increased LNC in NIL(*CAR8*) likely increases the cytochrome *b*_6_*/f*, but could also the photosystems, quinones, and plastocyanin components of whole chain-electron transport. Enhanced Calvin cycle protein may also contribute to higher *A* should it share in the limitation of *J*_max_ (Raines, 2011). Enzymes of starch and sucrose synthesis probably do not, as the CO_2_ responsiveness apparent in the *A/C*_i_ curve at 370 μmol mol^−1^ is much greater than would be expected under a P_i_ regeneration limitation (Sage, 1990). The higher LNC might be explained by the higher net accumulation of aboveground nitrogen and/or the higher rate of distribution of nitrogen to leaves (Mae and Ohira, 1981). This should be elucidated in future study. It is known that *HAP3* genes are associated with chloroplast biosynthesis and photosynthesis. In rice, an RNA interference construct silencing *OsHAP3A, OsHAP3B*, and *OsHAP3C* resulted in reduced expression of nuclear-encoded photosynthesis genes and degenerated chloroplast (Miyoshi et al., 2003). Recently, Alam et al. (2015) showed the overexpression of *OsHAP2E* increased the leaf chlorophyll content and *A* in rice. These suggest that HAP members redundantly affect the leaf photosynthesis in rice. It is also reported that the overexpression of *TaNF-YB3*, a member of *HAP3*, led to increases in the leaf chlorophyll content and photosynthesis in wheat (*Triticum aestivum*, Stephenson et al., 2011). These reports imply the association to the increased *J*_max_ in this study.

We then considered the second hypothesis that higher *A* results from enhancement of CO_2_ diffusion from air into leaves. NIL(*CAR8*) showed higher *g*_s_ than “Koshihikari” at full heading stage (Table 1). While much of the *g*_s_ response could reflect the regulation of *g*_s_ to track *A* (Wong et al., 1979), there was a slight increases in *C*_i_ and *C*_i_/*C*_a_ ratio in NIL(*CAR8*) relative to “Koshihikari” (Table 1). The higher *C*_i_/*C*_a_ in NIL(*CAR8*) demonstrate a greater proportional increase in *g*_s_ than *A*, such that the stomatal control over *A* has been relaxed. The 3% higher *C*_i_ in NIL(*CAR8*) than “Koshihikari” at the ambient CO_2_ concentration corresponds to increase of 1.5 μmol m^−2^ s^−1^ of *A* calculated from the *A*/*C*_i_ curve. These results indicate that *CAR8* enhances *g*_s_ independently of *A*.

In rice, *g*_s_ is determined by stomatal density, pore length, and aperture (Maruyama and Tajima, 1990; Ohsumi et al., 2007). Our results show that *CAR8* increases *g*_s_ by increasing stomatal aperture rather than stomatal density (Figure 5). The increase in stomatal density may also increase *g*_s_ in rice because stomatal density is higher in “Habataki” than in “Koshihikari” (Figure 5). This indicates that “Habataki” has alleles that enhance stomatal density, and a combination of these alleles and *CAR8* may further enhance *g*_s_.

Stomatal conductance responds to changes in plant water status (Schulze and Hall, 1982), and several studies have shown that it is closely related to *C*_p_ (Meinzer and Grantz, 1990; Hirasawa and Ishihara, 1992; Hubbard et al., 2001; Cochard et al., 2002; Brodribb et al., 2007). NIL(*CAR8*) had higher *C*_p_ than “Koshihikari” due to higher *L*_p_ (Table 3). We also found that Ψ_l_ in NIL(*CAR8*) was similar to that of “Koshihikari” while *g*_s_ in NIL(*CAR8*) remained high regardless of VPD conditions (Figure S2). This suggests that the higher water uptake of the root in NIL(*CAR8*) keeps Ψ_l_ high and decreases the risk of water stress even though *T* in NIL(*CAR8*) is significantly higher than “Koshihikari.” Therefore, the higher *g*_s_ in NIL(*CAR8*) would be partially explained by the higher *L*_p_. In contrast, Sakurai-Ishikawa et al. (2011) suggested the increase of water demand of shoots enhances root hydraulic conductivity via increase in gene expression of several aquaporins in the plasma membrane intrinsic protein family. This might explain the concomitant increases of *g*_s_ and *L*_p_ in NIL(CAR8). To our knowledge, there has been no report that shows association between HAPs genes and stomatal conductance. The identification of molecular network of *CAR8* would help to understand the regulations of stomatal conductance in rice. Kanemura et al. (2007) reported a weak negative relationship between g_s_ of flag leaves and days to heading using the rice diversity research set of germplasm. This suggests that flowering time affects photosynthesis of flag leaves and the allelic variation of *CAR8* would explain in part the natural variation of photosynthesis. This also implies the necessity to determine the association between flowering genes and photosynthesis, comprehensively.

The final grain yield in NIL(*CAR8*) was inferior to that in “Koshihikari” (Figure S3). This might be resulted from the short growth duration due to the “Habataki” allele of *CAR8* gene. To enhance the grain yield in rice, we should extend the growth duration of NIL(*CAR8*) by adding genes which delay heading date or modifying the growth conditions such as planting time.

In conclusion, the “Habataki” allele of *CAR8* associates to LNC under the 12-L pot condition. The higher LNC in NIL(*CAR8*), which relates to the higher RuBP regeneration rate, would mainly explain the enhanced *A* of the flag leaves. The “Habataki” allele of *CAR8* also associates hydraulic conductivity and hydraulic conductance at full heading stage under the 3-L pot condition. This could allow for a higher *g*_s_ in NIL(*CAR8*), which would partially explain the enhanced *A*. The fine mapping suggested that *CAR8* encodes a putative OsHAP3 subunit of a CCAAT-box-binding transcription factor and is identical to *DTH8/Ghd8/LHD1*, which has been reported to regulate flowering date. Identification of its molecular function would help understanding the association between photosynthesis and flowering and demonstrate specific genetic mechanisms that can be exploited to improve photosynthesis in rice and potentially other crops.

## Author contributions

SA, TY, RS, and JY designed the experiments. SA, KY, UY, and JS performed the experiments. TT built the stomata counting system. SA, TO, RS, TH, and JY wrote the manuscript.

## Funding

This work was supported in part by Grants-in-Aid from the Japan Society for the Promotion of Science (Postdoctoral Fellowship to SA), Japan Science and Technology Agency, Precursory Research for Embryonic Science and Technology to SA, the Ministry of Agriculture, Forestry and Fisheries of Japan (Genomics-based for Agricultural Innovation, RBS-2006 to TH), and the Institute of Global Innovation Research in TUAT to SA and TH.

### Conflict of interest statement

The authors declare that the research was conducted in the absence of any commercial or financial relationships that could be construed as a potential conflict of interest. The reviewer AS and handling Editor declared their shared affiliation, and the handling Editor states that the process nevertheless met the standards of a fair and objective review.

## Abbreviations

*A*: CO_2_ assimilation rate
*C*_a_: ambient CO_2_ concentration
*CAR8*: *carbon assimilation rate 8*
*C*_i_: intercellular CO_2_ concentration
*C*_p_: hydraulic conductance from roots to leaves
dCAPS: derived cleaved amplified polymorphic sequence
DAS: days after sowing
*DTH8*: *days to heading 8*
*Ehd1*: *Early heading date1*
*g*_s_: stomatal conductance
Γ^*^: CO_2_ compensation point in the absence of day respiration
*Ghd8*: *grain number plant height and heading date 8*
*GPS*: *Green for Photosynthesis*
HAP: heme activator protein
*Hd1*: *Heading date 1*
*Hd3a*: *Heading date 3a*
InDel: insertion-deletion
*J*_max_: maximum rate of electron transport
*K*_c_: Michaelis constants for CO_2_
*K*_o_: Michaelis constants for O_2_
*LHD1*: *Late Heading Date 1*
*L*_p_: hydraulic conductivity
LNC_a_: leaf nitrogen content per leaf area
LNC_w_: leaf nitrogen content per leaf dry weight
NIL: near-isogenic line
*O*: intercellular O_2_ concentration
PPFD: Photosynthetic photon flux density
Ψ_l_: leaf water potential
QTL: quantitative trait locus
*R*_d_: day respiration rate
Rubisco: ribulose-1,5 bisphosphate carboxylase/oxygenase
RuBP: ribulose 1,5-bisphosphate
*S*_r_: root surface area
*T*: transpiration rate
*V*_cmax_: maximum rate of RuBP carboxylation
VPD: vapor pressure deficit.
