# Supplementary material for: Fine Mapping of Carbon Assimilation Rate 8, a Quantitative Trait Locus for Flag Leaf Nitrogen Content, Stomatal Conductance and Photosynthesis in Rice
Source: Front Plant Sci. 2017 Jan 31;8:60. doi: 10.3389/fpls.2017.00060 (PMC5282472; doi:10.3389/fpls.2017.00060)
Supplement: Supplementary file 1 [file Presentation1.PDF]

Supplementary Data

Fine Mapping of *Carbon Assimilation Rate 8,* a Quantitative Trait Locus for Flag Leaf Nitrogen Content, Stomatal Conductance and Photosynthesis in Rice

Shunsuke Adachi, Kazuaki Yoshikawa, Utako Yamanouchi, Takanari Tanabata, Jian Sun, Taiichiro Ookawa, Toshio Yamamoto, Rowan Sage, Tadashi Hirasawa, Junichi Yonemaru

Table S1. Primer sequences used for the mapping of *CAR8*.

Name	Upper primer (5’-3’)	Lower primer (5’-3’)
RM6999	TTATCTGGGATCCATCGAGC	GTGAATTTCCTTGGAGGGAC
InDel8-12	AAACATATAGAAGAACTGTTTGTGTCA	GGACGAAACCCTAATCTTCACTT
dCAPs8-1	TCCGTTCTACTCTCTCCATT	TTTGATTTAGAACCAACCTAAGATGA
InDel8-43	CGGAAAACGAGACAATCACCCCT	AACGCAGCTGCAATTAAATAAAA
InDel8-22	ACAAGCCTAGCTACTTGGAACAT	CACTGACTCCAATTGAAATGACA
InDel8-26	GCCCTAATACATGATGCCTCTAA	AAATTGTAATATTGACGCGGTTG
RM22506	AATGACCCATTATGCGGCAAAGC	CCTTTGGCGCACCAACTAAGAGTACC
RM22529	TGCGAGTATTTAACTCACCCATCC	CTTGCCTCACAAGATCCAAACC

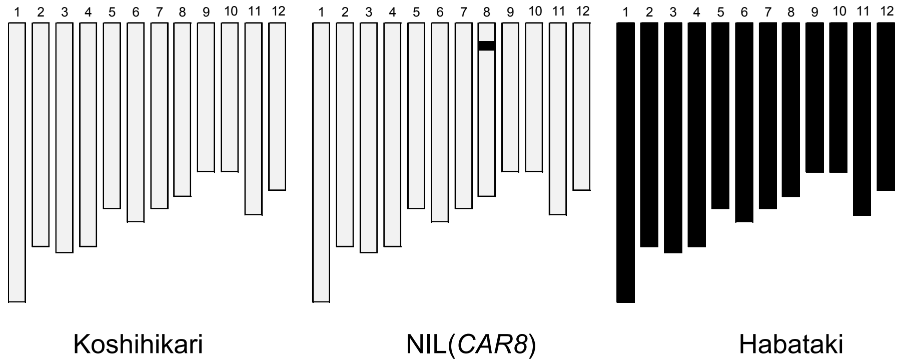

Figure S1. Graphical genotypes of 'Koshihikari', NIL(*CAR8*), and 'Habataki'. NIL(*CAR8*) has a single chromosome segment from 'Habataki' in the genetic background of 'Koshihikari' that is represented in the black region. NIL(*CAR8*) is the same as Line-11 in Figure 1. The length of the substituted region in NIL(*CAR8*) was approximately 1.0 Mb.

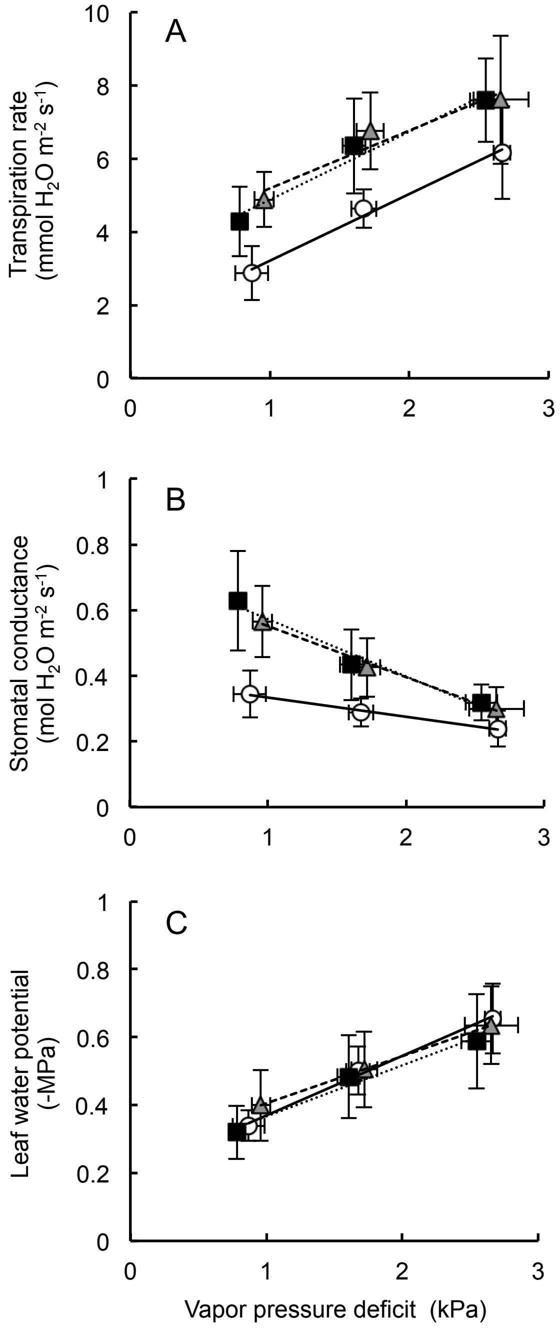

Figure S2. The response of transpiration rate (A), stomatal conductance (B), and leaf water potential (C) to the change of vapor pressure deficit. Open circle, shaded triangle and filled square represents 'Koshihikari', NIL(*CAR8*) and 'Habataki', respectively. Values are mean ± SD (*n* = 8).

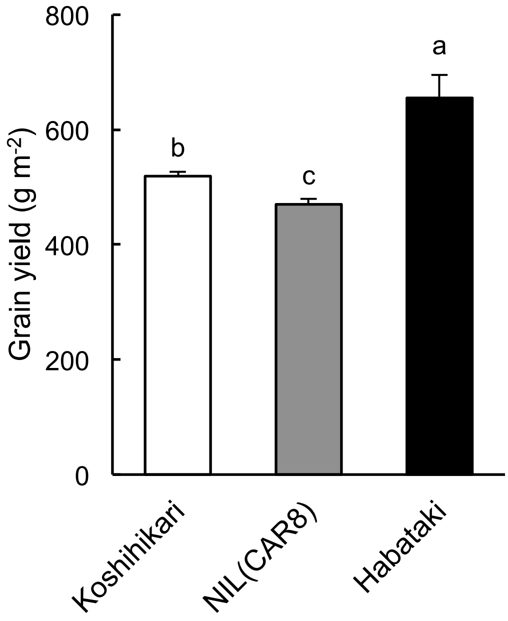

Figure S3. The grain yield of the plants grown in paddy field. The plants in area of approximately 1.4 m^2 for each replicate were harvested. Grain yield was determined as a yield of brown rice. Fully ripened grains were selected by sieving through 1.8-mm mesh and adjusted to a moisture content of 14.5%. Values are mean ± SD (n = 4).
